# The role of the innate immune response regulatory gene ABCF1 in mammalian embryogenesis and development

**DOI:** 10.1371/journal.pone.0175918

**Published:** 2017-05-19

**Authors:** Sara M. Wilcox, Hitesh Arora, Lonna Munro, Jian Xin, Franz Fenninger, Laura A. Johnson, Cheryl G. Pfeifer, Kyung Bok Choi, Juan Hou, Pamela A. Hoodless, Wilfred A. Jefferies

**Affiliations:** 1 The Michael Smith Laboratories, The University of British Columbia, Vancouver, British Columbia, Canada; 2 Department of Microbiology & Immunology, The University of British Columbia, Vancouver, British Columbia, Canada; 3 Department of Zoology, The University of British Columbia, Vancouver, British Columbia, Canada; 4 Terry Fox Laboratory, BC Cancer Agency, Vancouver, British Columbia, Canada; 5 Department of Developmental and Cell Biology, The University of British Columbia, Vancouver, British Columbia, Canada; 6 Department of Medical Genetics, The University of British Columbia, Vancouver, British Columbia, Canada; 7 Centre for Blood Research, The University of British Columbia, Vancouver, British Columbia, Canada; 8 Djavad Mowafaghian Centre for Brain Health, The University of British Columbia, Vancouver, British Columbia, Canada; University of Connecticut, UNITED STATES

## Abstract

ABCF1 is an ABC transporter family protein that has been shown to regulate innate immune response and is a risk gene for autoimmune pancreatitis and arthritis. Unlike other members of ABC transporter family, ABCF1 lacks trans-membrane domains and is thought to function in translation initiation through an interaction with eukaryotic translation initiation factor 2 (eIF2). To study ABCF1 expression and function in development and disease, we used a single gene trap insertion in the *Abcf1* gene in murine embryonic stem cells (ES cells) that allowed lineage tracing of the endogenous *Abcf1* promoter by following the expression of a β-galactosidase reporter gene. From the ES cells, heterozygous mice (*Abcf1+/-*) were produced. No live born *Abcf1-/-* progeny were ever generated, and the lethality was not mouse strain-specific. Thus, we have determined that *Abcf1* is an essential gene in development. *Abcf1-/-* mice were found to be embryonic lethal at 3.5 days post coitum (dpc), while *Abcf1+/-* mice appeared developmentally normal. *Abcf1+/-* mice were fertile and showed no significant differences in their anatomy when compared with their wild type littermates. The *Abcf1* promoter was found to be active in all organs in adult mice, but varies in levels of expression in specific cell types within tissues. Furthermore, we observed high promoter activity in the blastocysts and embryos. Overall, *Abcf1* expression in embryos is required for development and its expression in adults was highly correlated with actively proliferating and differentiating cell types.

## Introduction

The ATP binding cassette (ABC) super-family of proteins are present in all phyla and harness the energy from the hydrolysis of ATP in order to transport substrates across cellular membranes and power cellular machinery [1, 2]. All members of the ABC super-family contain conserved Walker motifs along with a signature ABC motif, which are responsible for the hydrolysis of ATP [3]. Members of the ABC super-family can be grouped into three categories: importers, exporters, or the third category, which are involved in DNA repair and translation and lack trans-membrane domains [1]. ABCF1 (also called ABC50), falls into this latter category, as it lacks trans-membrane domains [4]. ABCF1 was initially identified as a protein that was up-regulated in synoviocytes in the presence of TNFα in patients with rheumatoid arthritis [4]. It has since been found to be essential for regulating innate response to cytosolic DNA and retroviral infection [5]. Polymorphism in the *ABCF1* gene has also been associated with autoimmune pancreatitis in the Japanese population [6]. Its genetic sequence indicates that it may be homologous to the yeast ABC transporter GCN20 [7, 8]. GCN20, along with GCN1, is thought to coordinate the trafficking of uncharged tRNAs to GCN2 during the process of translation elongation [9]. GCN2 is a protein kinase, which is activated by binding to uncharged tRNA [7]. Upon activation, GCN2 phosphorylates the eukaryotic initiation factor 2 α subunit (eIF2a), thus reducing protein synthesis in times of amino acid starvation [9]. This process regulates protein synthesis by coupling the availability of amino acids to the control of eIF2α by GCN2 [7]. Like GCN20, ABCF1 was found to be important in translation initiation in an *in vitro* siRNA model [10]. However, the protein sequence of ABCF1 differs from that of GCN20 at the N-terminus, having only 20% identity and 30% similarity to GCN20 in this region [10]. The N-terminal sequence of GCN20 is responsible for binding to GCN1 and complementing the function of GCN2, thus indicating that ABCF1 has a function that is distinct from that of GCN20 [7, 11].

ABCF1 was found to directly associate with eIF2 and to markedly enhance binding of methionyl tRNA (Met-tRNA) to eIF2 [10, 12]. The N-terminal sequence of ABCF1 was found to be important for its interaction with eIF2, particularly the first 42 residues [13]. When *ABCF1* was knocked down in Hela cells through siRNA, both cap-dependent and cap-independent translation of reporter genes was inhibited [7]. Mutations in either of the ATP-binding Walker motifs were found to reduce reporter gene cap-dependent translation in Hela cells, with Walker B mutants showing greater inhibition [7]. Interestingly, although ABCF1 had previously been shown to be involved with translation initiation, its role in development has not been studied. To date, the only information regarding the *ABCF1* promoter and its regulation show that it is regulated by TNFα [4]. To examine the expression of ABCF1 in physiology, we generated an *Abcf1* knockout mouse model from embryonic stem cells that contained a gene-trap cassette in the *Abcf1* gene. With this approach [14], genes are “trapped” by the random insertion of a gene trap vector into an intronic sequence of the endogenous gene [15]. The gene trap vector contains splice acceptor sites with reporter genes, selectable markers, and polyadenylation sequences. Splice acceptor sequences within the gene trap vector mimic an endogenous intron-exon boundary, and polyadenylation sequences result in a truncated mRNA fused to the trap vector sequence [15]. Therefore, the endogenous promoter drives the expression of the gene trap vector that produces a fusion transcript [15]resulting in a truncated or mutant cellular protein [15]. The β-galactosidase (*β-gal*) gene from *Escherichia coli* is a common reporter gene that is used in gene trapping [16]. Translation of this mRNA results in the production of a truncated protein fused to β-gal [15, 17]. β-gal expression is quickly and easily detected *in situ* by staining cells or tissues [16]. The expression of β-gal in gene trap models has previously been shown to be proportional to the endogenous promoter activity of the trapped gene if the fusion proteins are expressed [16]. Here, we describe a mouse that was generated with a single gene trap vector inserted into the intronic region 31bp downstream of exon 7 of *Abcf*1. We have determined that no live *Abcf1-/-* mice could be generated from *Abcf1+/-* matings and that the embryos of *Abcf1-/-* mice are lethal shortly after implantation. The gene-trap cassette insertion results in the production of a truncated fusion protein containing the N-terminal region of the *Abcf1* gene (the first seven exons) fused to a β-galactosidase and neomycin phosphotransferase II fusion gene (β-geo). This gene-trap insertion allows the expression of β-geo to be under the control of the endogenous *Abcf1* promoter. Since β-geo expression can be visualized through staining, this system allows us to examine the physiological *Abcf1* promoter activity in different tissues at different stages of development. Here, we find that its expression in embryos and adults is highly correlated with actively proliferating and differentiating cell types, consistent with its role in translation.

## Materials and methods

### Genotyping

The ES cell line XK097 (https://www.mmrrc.org/catalog/cellLineSDS.php?mmrrc_id=9019) from Bay Genomics (http://www.informatics.jax.org/allele/key/343209), strain 129P2/OlaHsd, contains a gene trap vector in the *Abcf1* gene. Previously, 5’-RACE (5’ rapid amplification of cDNA ends) and DNA sequencing was used to identify the trapped gene using primer sequences unique to the gene trap vector [18]. We re-confirmed the presence of the vector sequence in the XK097 ES cells using RT-PCR. ES cells were grown in DMEM media supplemented with 2 mM L-glutamine, 1 mM sodium pyruvate, 1 mM non-essential amino acids, 100 U/ml penicillin/streptomycin, 2-mercaptoethanol and 15% FBS. They were then washed extensively in PBS to remove media, and RNA was extracted using Trizol reagent (Invitrogen). cDNA was created using Superscript II (Invitrogen) according to the manufacturer’s conditions. PCR was used to confirm the presence of the vector sequence using primers: forward 5’-CAGCCCGCACCTCTC-3’ (int1-f) and reverse 5’-GACAGTATCGGCCTCAGGAAGATCG-3’ (Bay-cDNA). The control primers for the wild type allele were forward 5’-GGCTCAGGAGTAAAAAGGGAA-3’ (Ex7-f) and reverse 5’-CCTGCTCAGCCTCCTTTTTGT-3’ (Ex-8-r). The reactions were performed on a TProfessional TRIO thermal cycler (Biometra) using the following conditions: 95°C for 5 min, followed by 40 cycles of 95°C for 30 s, 60°C for 30 s and 72°C for 30 s followed by a 10 min 72°C elongation step. Genotyping of the XK097 cell line, blastocyst embryos, and the *Abcf1* knockout mice was performed by PCR using Platinum Taq polymerase (Invitrogen). The genomic DNA genotypes were determined by using two different PCRs. The first (1250 bp) which spanned the insertion site included primer int1-f, which amplified the intronic sequence 5’ of exon 7 and Bay-cDNA, which recognized a sequence within the β- galactosidase reporter gene. The second PCR (1304 bp), which amplified the wild-type allele, included primers for exon 3 5’-AAGAGAGGAAATGGGGCAGT-3’ (Ex3-f) and exon 8 (Ex8-r). The reactions were performed on a TProfessional TRIO thermal cycler (Biometra). The conditions for both reactions were 95°C for 5 min, followed by 40 cycles of 95°C for 30 s, 55°C for 30 s and 72°C for 1 min 30 s, followed by a 10 min 72°C elongation step. DNA from the reaction was separated and visualized by agarose gel electrophoresis (1% gel) and Sybr-Safe staining (Invitrogen).

For genomic DNA isolation from blastocysts, the blastocysts were washed several times in M2 media (Sigma Aldrich), then transferred to PBS for one wash before placing them individually in Eppendorf tubes containing 10 μL blastocyst lysis buffer solution (50 mM Tris-HCL (pH 8.0), 0.5% Triton X-100 and 200 μg proteinase K (Fermentas)) [19, 20]. Blastocysts were then freeze-thawed and incubated for 40 min at 55°C. Proteinase K was inactivated by a 10 min incubation at 95°C. 1 μL of template was used per reaction. For genomic DNA isolation from fetuses, we first set up timed matings between female *Abcf1*^*+/-*^ mice and *Abcf1*^*+/-*^ males. At various times of gestation, the female mice were sacrificed with CO_2_ asphyxiation, and their uteri were removed. Embryos were separated from their yolk sacs, and genomic DNA was extracted using the blastocyst lysis buffer and protocol described above.

For DNA isolation from adult mice, ear clips were placed in 20 μL of lysis buffer (50 mM Tris HCl (pH 8.0), 2 mM NaCl, 10 mM EDTA, 1% SDS and 1 mg/mL proteinase K (Fermentas)) and incubated for 60 min at 55°C. We then added 300 μL of milliQ water to each sample, which was then incubated for 10 min at 95°C to inactivate the proteinase K. Subsequently, 700 μL of milliQ water was added to each sample, and 4 μL of this template was used in subsequent reactions.

### Creation of the ABCF1 deficient XK097 mice

The XK097 ES cells (strain 129P2/OlaHsd; chinchilla, white, agouti) were microinjected into C57BL/6 (black) blastocysts in order to create chimeric mice. Blastocysts were obtained by setting up timed matings between C57Bl/6 male and female mice and checking for copulatory plugs the following day. Blastocysts were flushed out of the uterine horns of the plugged females at 3.5 dpc and were used for microinjection. ICR mice were purchased from Harlan Laboratories, and pseudopregnancy was induced by setting up females in estrus with vasectomized males and checking the following day for copulatory plugs. The injected blastocysts were implanted into the uterine horn of a 2.5 days post-coitum (dpc) pseudopregnant ICR female mice. Pups were then born three weeks later, and chimeras were identified by coat color. The highly chimeric males were then back-crossed with C57BL/6 females (Jackson Laboratories). Progeny from these mice were then further genotyped and back-crossed 12 generations onto the inbred C57BL/6 line, the inbred Balb/C line, and the out-crossed ICR line. Animals were fed a standard diet and were routinely screened for pathogens. Euthanasia and animal sacrifice was performed by CO_2_ inhalation.

Mice were housed in the mouse facility at the Biomedical Research Centre (University of British Columbia: UBC) and subsequently at the Centre for Disease Modeling (UBC) where they were maintained according to the protocols and procedures of the Canadian Council on Animal Care (CCAC). The University of British Columbia Animal Care Committee specifically approved the work done in this study, under the ethic protocol numbers A07-0270 and A13-0079.

### Collection of mouse blastocysts

To isolate blastocysts, female *Abcf1*^*+/-*^ mice were first superovulated (given an inter-peritoneal (ip) injection of 5 IU Pregnant Mare Serum (PMS), followed 46 hours later with ip injection of 5 IU Human Chorionic Gonadotropin (hCG), and then mated with *Abcf1*^*+/-*^ male studs. Females were sacrificed using CO_2_ asphyxiation 3.5 dpc, and their uteri were dissected out and flushed with M2 media (Sigma Aldrich) into 6 mm plates (Falcon-Becton Dickinson).

### X-Gal staining and histology

The reporter gene used in the gene-trap vector was β-geo. In order to assess the β-geo protein activity in mouse embryos and whole organs, tissues were excised and fixed in X-Gal fixation buffer (0.1 M phosphate buffer, pH 7.3 (3.74g NaH_2_PO_4_:H_2_0 FW = 137.99; 10.35 g Na_2_HPO_4_, FW = 141.96; in 1 L H_2_O), 5 mM EGTA, 2 mM MgCl_2,_ and 0.2% gluteraldehyde) for 15–60 min depending on the size of the tissue. They were then washed three times for 5 min with X-Gal wash buffer (0.1 M phosphate buffer, pH 7.3, 2 mM MgCl_2_) and stained overnight at 37°C in X-gal staining buffer (0.1 M phosphate buffer, pH 7.3, 5 mM K_4_Fe(CN)_6_:3H_2_O, 5 mM K_3_Fe(CN)_6_, 2 mM MgCl_2_, 1 mg/mL X-Gal (5-bromo-4-chloro-3-indolyl-β-D-galactopyranoside)). Stained tissues were then washed three times with X-Gal wash buffer. Sectioned embryos and tissues were fixed, stained, and washed using the same protocol as above. A fixation time of 15 min was used for cut sections. Sections were taken through entire embryos or tissue from multiple mice. After the final wash step, tissues were cryoprotected by incubation overnight at 4°C in a 20% sucrose solution. After incubation, tissues were immersed in Tissue-Tek O.C.T compound (VWR Scientific) and snap-frozen in liquid nitrogen, then stored at -80°C until sectioning. Sections of tissues, 12–16 μm thick, were cut onto Superfrost Plus Micro slides (VWR Scientific), and slides were then washed by carefully dipping them several times in X-gal wash buffer. For larger tissue sections, sections were first cut, washed, and then stained overnight at 37°C in X-gal staining buffer. Slides were counter-stained with Nuclear fast red (Sigma Aldrich). Sections were dehydrated, then mounted in Xylene-based mounting medium (Fisher-Scientific). In order to ensure the specificity of β-geo activity, tissues from wild type littermate controls were stained in tandem. To isolate blastocysts for staining, females were first superovulated (as above) to maximize the number of blastocysts recovered. Females were sacrificed by CO_2_ asphyxiation at varying times from 3.5dpc to 13.5 dpc, and their uteri were dissected out and flushed with M2 media. Individual blastocysts were isolated and washed several times in M2 media, then placed into fixation buffer for 2 min. The blastocysts were then washed and placed in X-gal staining buffer. The blastocyst staining was apparent after 1 h, after which the cells were washed once with PBS and analyzed by microscopy.

### Image quantification

Image analysis algorithms were developed using MatLab 7 (MathWorks, Apple Hill Drive, MA, USA). To quantify X-gal staining, readouts from the red, green and blue (RGB) channels of each image were obtained and two threshold values were defined for each organ respectively. The value of each RGB channel of every pixel had to be below an upper threshold to remove background in every image. Pixels with RGB values below the lower thresholds were quantified as X-gal positive. The percentage of X-gal staining was then calculated by diving the number of X-gal positive pixels by the number of total pixels of the image, corrected by the number of background pixel. For each organ n = 7 images were analyzed. Images of wild type mice (*Abcf*1+/+) without the ß-gal transgene, were then analyzed for each organ as a negative control. Percentage 1–10% is characterized as light staining, 10–40% as intermediate staining and greater than 40% as intense staining.

### Microscopy

All Images 10× and above were taken using an Olympus FV1000MPE microscope and Zeiss Pascal Excite (Laser Scanning Confocal Microscope), and images below 10× were taken with an Olympus Stereomicroscope at UBC Bioimaging Facility, University of British Columbia, Vancouver, Canada.

## Results

### The ES cell line XK097 contains a gene trap insertion in the *Abcf1* gene

**Fig 1:**
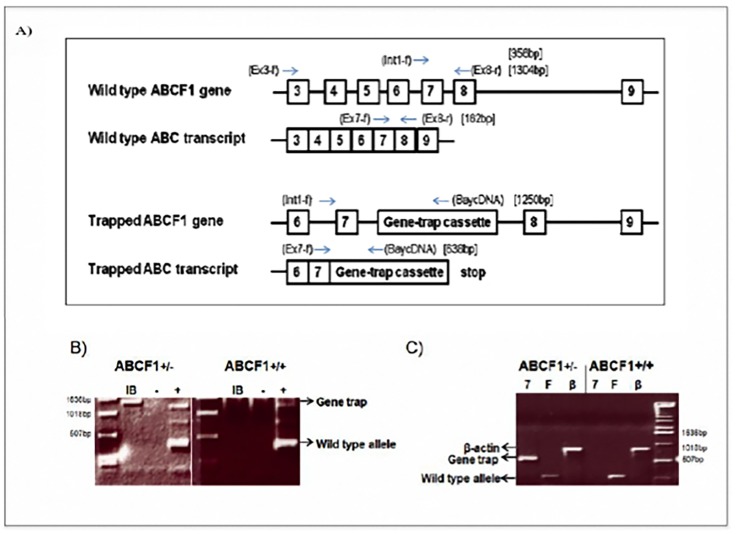
The ES cell line XK097 contains a gene trap insertion in the *Abcf1* gene. A) The genomic and mRNA location of the Bay Genomics pGT0Lxf vector (gene trap) on chromosome 17 corresponding to the *Abcf1* locus in the XK097 ES cell line. The locations and names of the primers used for screening are in round brackets. The size of the PCR product is indicated in square brackets. B) The ES cell line, XK097, contains the gene trap sequence between exon 7 and 8 in the *Abcf1* gene. Genomic DNA analysis of the XK097 cell line (left panel) compared with a wild type ES cell line (right panel). “IB” (Int1-f and Bay cDNA (1250bp)) primer combination indicates the presence of the gene trap cassette in the *Abcf1* locus. “**-**” is the negative water alone control and “**+**” is the positive control (Rps15 (350bp)) for the PCR. C) RT-PCR of the ES cell line, XK097, produces a transcript containing exon 7 and the Bay Genomics vector sequence. RT-PCR analysis of the XK097 cell line (*Abcf1*^*+/-*^; left) compared with a wild type ES cell line (*Abcf1*^*+/+*^; right). 7 = Ex7-f-Bay-cDNA primers, F = Ex7-Ex8 primers, β = *β-actin* primers.

The gene trap in XK097 was inserted into the intronic sequence between exon 7 and exon 8, thus creating a gene fusion containing exons 1–7::β-geo (Fig 1A). The primer set that produced the most consistent positive results (Int1-f and Bay cDNA) was then used to generate a fragment, which was further cloned and sequenced (Fig 1B). This 1.25 kb sequence confirmed that the gene trap vector had inserted into the intronic sequence 31 bp downstream of exon 7. To differentiate the trapped from wild type genomic sequence, another set of primers (Ex3f-Ex8r) was generated to produce a 1.3 kb product that spanned the insertion site.

RT-PCR was used on the XK097 cell line to confirm that the gene trap was functional and that the insertion was between exon 7 and exon 8 (Fig 1C). Transcription is engineered to end after the reporter gene, and the ATP binding cassettes begin translation at exon 10, so the resulting fusion protein is most likely non-functional with respect to its ATP hydrolysis function. We then used this cell line to produce a chimeric male mouse, which was used to create *Abcf1*^*+/-*^ knockout mice. These mice were then backcrossed 12 generations to both C57BL/6 and Balb/C females.

### *Abcf1* homozygous knockout mice produced from the XKO97 cell line are embryonic lethal

Mice positive for one copy of the gene trap cassette (*Abcf1*^*+/-*^) were mated to produce homozygous knockout mice. Ear clips were taken from 227 progeny and genotyped by PCR. None of the pups were positive for the gene trap cassette alone. *Abcf1*^*+/-*^ males were also backcrossed several generations onto the Balb/C strain. After several generations of backcrossing, the heterozygous pups were inter-crossed. No adult *Abcf1*^*-/-*^ pups were ever identified in the Balb/C strain, indicating that the lethality associated with the lack of *Abcf1* was not strain-specific. The ratio of wild-type to heterozygous mice was approximately 1:2, which is the expected ratio if the gene trap is embryonic lethal. Female *Abcf1*^*+/-*^ mice were then timed mated to *Abcf1*^*+/-*^ studs and dissected at 6.5 to 10 dpc. Embryos could not be recovered in approximately 1 out of 4 placentae, which indicated that they had been resorbed. PCR genotyping also confirmed that none of the embryos were homozygous knockouts, and the ratio of wild-type to heterozygous embryos was approximately 1:2. Female *Abcf1*^*+/-*^ mice were superovulated, then mated and sacrificed at 3.5 dpc. Uteri were dissected out and flushed. Individual blastocysts were isolated and genotyped by PCR. *ABCF1*^*-/-*^ blastocysts were identified at the expected 25% ratio (Table 1). Since empty placentae were present, embryos were likely non-viable shortly after implantation.

**Table 1:** Genotypes of the offspring from *Abcf1*^*+/-*^ inter-crosses show that approximately 25% of blastocysts are *Abcf1*^*-/-*^.

Genotype					
	ABCF1-/-	ABCF1+/-	ABCF1+/+	Resorbed	Total
3 Weeks+	0	143 (63%)	84 (37%)	N/A	227
E10.5 dpc	0	4 (40%)	4 (40%)	2 (20%)	10
E9.5 dpc	0	5 (56%)	2 (22%)	2 (22%)	9
E8.5 dpc	0	11 (55%)	4 (20%)	5 (25%)	20
E8 dpc	0	5 (46%)	3 (27%)	3 (27%)	11
E7.5 dpc	0	6 (60%)	3 (30%)	1 (10%)	10
E6.5 dpc	0	6 (54%)	3 (27%)	2 (18%)	11
E3.5 dpc	13 (21%)	33 (54%)	15 (25%)	N/A	61

Mice were either genotyped as 3 week old pups, or embryos from pregnant females were excised at different days post coitus (dpc). Genotyping was done by PCR. Values are displayed as number (%) for each time point.

### The *Abcf1* promoter is active during embryogenesis

**Fig 2:**
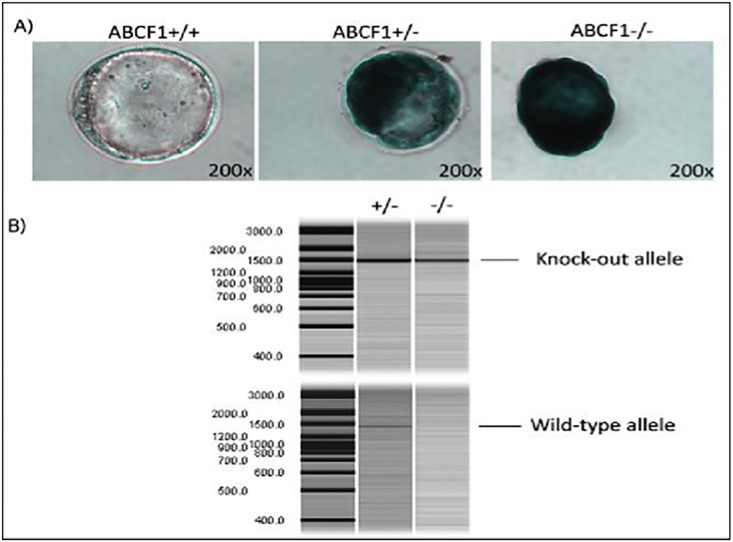
The *Abcf1* promoter is highly expressed during early embryogenesis. A) Blastocysts were isolated from 3.5 dpc female mice and stained with X-gal. Three separate phenotypes were observed: non-stained (*Abcf1*^*+/+*^*)*, intermediate-stained (*Abcf1*^*+/-*^*)* and darkly stained (*Abcf1*^*-/-*^), which directly corresponded to their genotypes and to the gene copy number of the β-geo reporter. The darkly stained blastocysts also appeared to be slightly oblong in shape compared with the medium-stained embryos and lacked a zona pellucida. Images were taken using a 20× lens. B) To distinguish between homozygous-knockout and heterozygous blastocysts, the blastocysts from A were genotyped by PCR to confirm the genotypes. The top image shows the presence of the knockout allele in both blastocysts. The bottom image (taken using an QIAxcel Capillary Electrophoresis machine at Biomedical Research Centre, UBC) shows the presence of the wild type allele only in the heterozygous blastocyst.

To examine the *Abcf1* promoter activity in blastocysts, blastocysts were isolated at 3.5 dpc and stained with X-gal to visualize the β-geo expression (Fig 2A). The genotypes of these blastocysts were later confirmed through PCR genotyping (Fig 2B). The endogenous *Abcf1* promoter drove the β-geo expression, therefore blue staining was indicative of *Abcf1* promoter activity. Given that *Abcf1*^*-/-*^ embryos were embryonic lethal shortly after implantation, two copies of the gene-trap were expressed in *Abcf1*^*-/-*^ blastocysts, while only one was expressed in *Abcf1*^*+/-*^ blastocysts. Staining was present throughout the blastocysts, with dark staining in the inner cell mass of both the *Abcf1*^*-/-*^ and *Abcf1*^*+/-*^ blastocysts and more diffuse staining in the blastocoels. The shape of the *Abcf1*^*-/-*^ blastocysts appeared to be more oblong, and they were more likely to lack a zona pellucida (Fig 2A).

**Fig 3:**
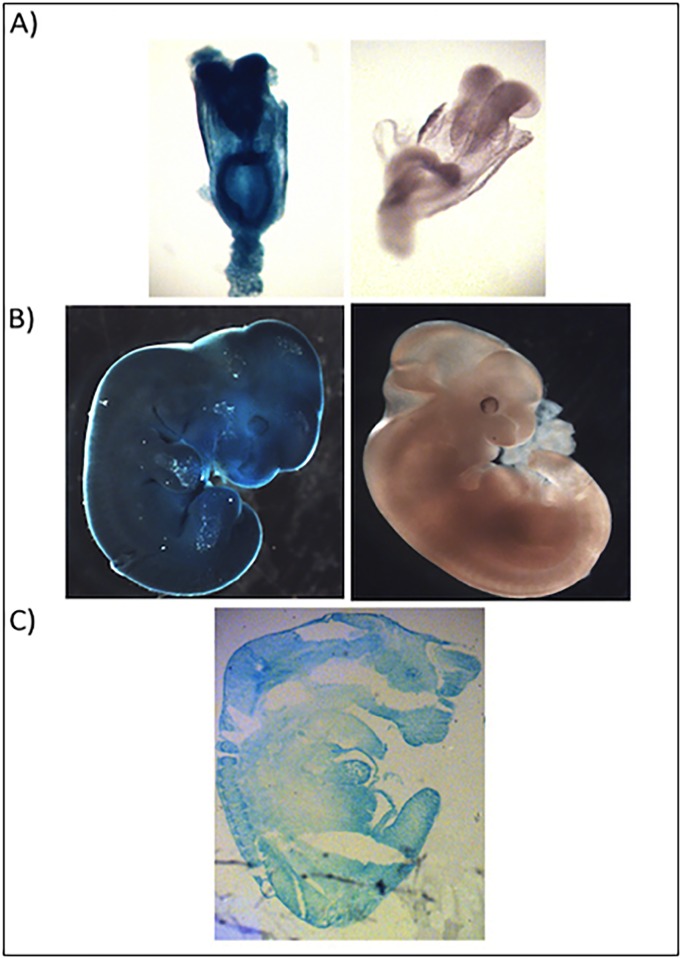
The *Abcf1* promoter is highly expressed throughout embryogenesis. A) 8.5 dpc and B) 11.5 dpc embryos were stained with X-gal, and *Abcf1*^*+/-*^ heterozygous (blue) embryos showed strong staining throughout the embryo, compared with *Abcf1*^*+/+*^ wild type (white) littermate controls, which showed no staining. C) An 11.5 dpc embryo was sectioned and showed that the promoter was active in all tissues. Images were taken using a 3.5× objective.

To investigate the *Abcf1* promoter activity in embryos, male *Abcf1*^*+/-*^ mice were timed mated to *Abcf1*^*+/-*^ females and their embryos were removed at various dpc. Whole embryos were stained and showed dark, diffuse staining throughout, indicating the *Abcf1* promoter was highly active in developing embryos (Fig 3A). These results were not surprising, as one functional copy of *Abcf1* appeared to be essential throughout embryogenesis. Cryosections of 11.5 dpc embryos confirmed that the *Abcf1* promoter was also highly active in all tissues of the developing embryo, and no precise lineage could be determined (Fig 3B and 3C). Very high activity was detected in the cranial nerves, optic cup, liver, heart, neural epithelium and dorsal root ganglion. The skin was darkly stained as well. All of these organs at this stage of development are known to be actively proliferating and differentiating [21].

### The *Abcf1* promoter is differentially expressed in adult tissue

Initially, to determine RNA expression of *Abcf1*, northern blot analysis was performed on adult mouse tissues (data not shown). Northern blot showed that *Abcf1* mRNA was expressed in all organs and tissues tested, particularly the spleen, thymus, testis, and ovaries. To investigate the physiologic Abcf1 promoter activity in adult mice, we examined X-gal staining in whole organs as well as cryosectioned tissues. Whole organ staining allowed us to identify general regions of interest, while sectioning allowed us to determine cell- and tissue-specific expression.

**Fig 4:**
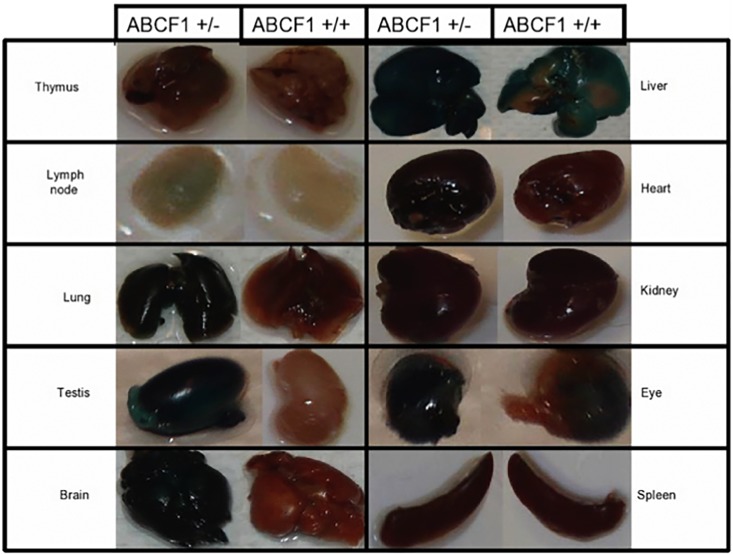
The *Abcf1* promoter is active in whole adult mouse organs. Whole organs were excised from *Abcf1*^*+/-*^ mice and their *Abcf1*^*+/+*^ littermate controls and stained with X-gal for 24 h. There was also some endogenous X-gal staining in the wild-type liver, testis, and thymus, so these organs were stained for 4–12 h, depending on the development of endogenous expression in the wild-type control.

Of the whole organs stained with X-gal, the staining was detectable in thymus, lymph node, lungs, testis, brain, liver, heart, kidney, eye and spleen (Fig 4). Nonspecific staining was unable to be eliminated in the whole mount livers, due to endogenous galactosidase activity, but the overall staining was not as intense as in the *Abcf1*^*+/-*^ livers. It has been reported that endogenous galactosidase activity levels in the liver may be mouse-strain specific [22, 23].

**Fig 5:**
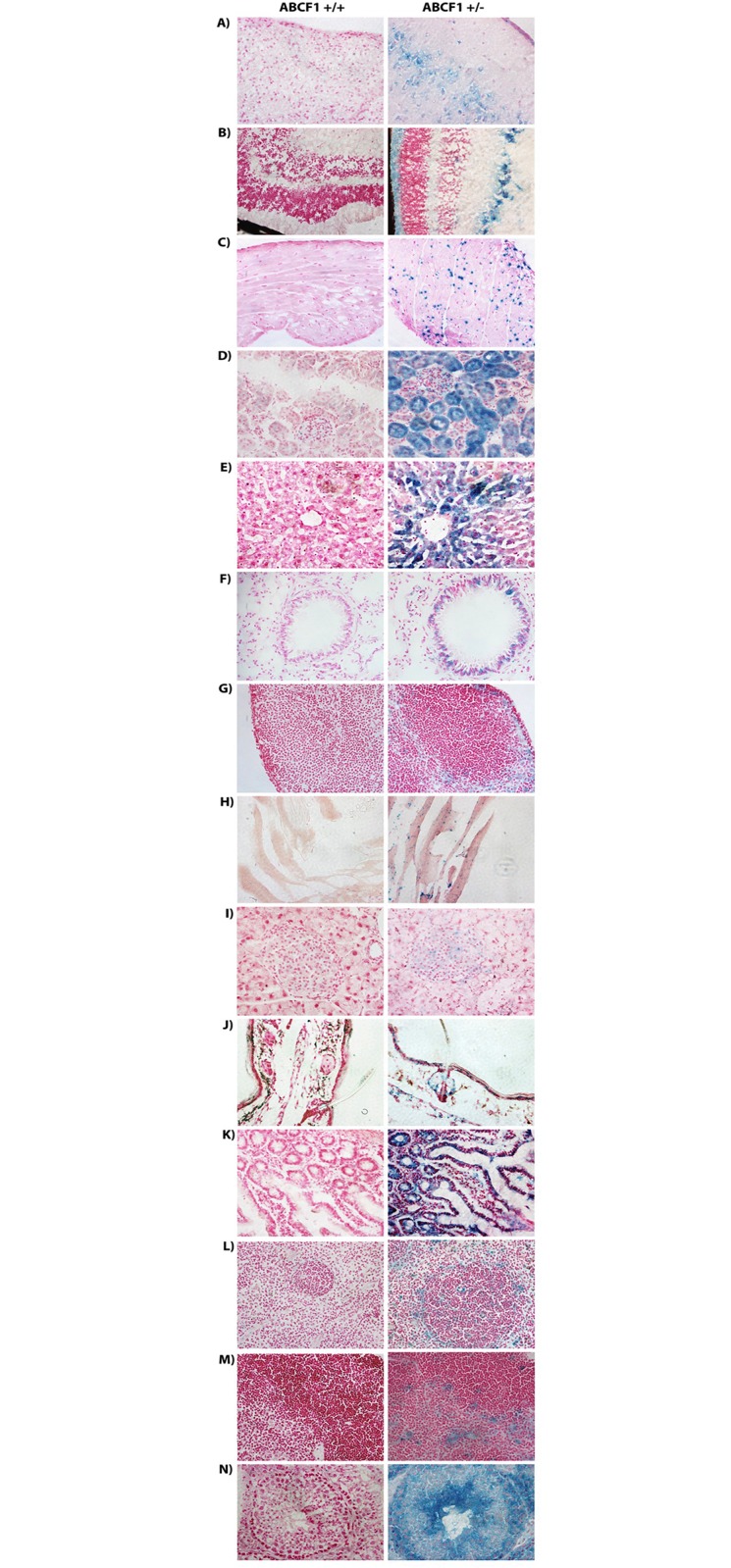
X-gal expression is variable in tissues from *Abcf1*^*+/-*^ adult mice. Variable *Abcf1* promoter activity was seen in A) Cerebral Cortex, B) Retina, C) Heart, D) Renal Cortex, E) Liver, F) Lung, G) Inguinal Lymph Node, H) Muscle, I) Pancreas, J) Skin, K) Small Intestine, L) Spleen, M) Thymus, and N) Testis. Tissues were stained with X-gal and counter-stained with nuclear fast red. Images were taken using the 40× objective.

**Fig 6:**
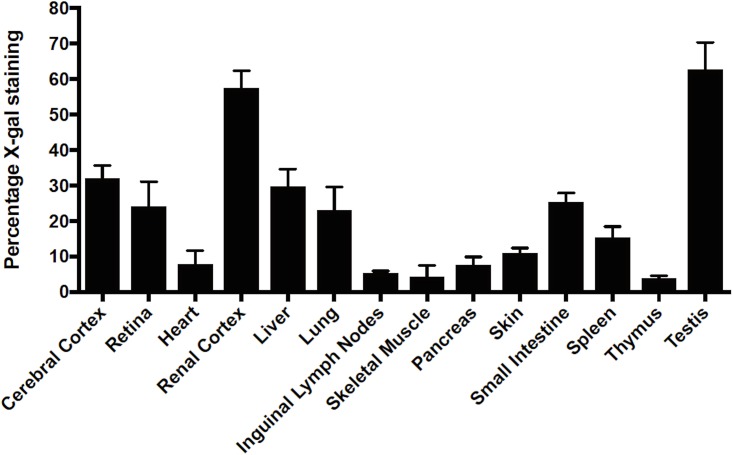
Quantitative representation of X-gal staining. Image quantification was done using MatLab (MathWorks, Apple Hill Drive, MA, USA), as described in the material and methods section. 7 animals each of control (*Abcf*1^+/+^; no β-geo) and heterozygotes (*Abcf*1^+/-^; expressing β-geo) were sacrificed, tissues were extracted and staining intensities were measured. The error bar represents the standard deviation. Percentage 1–10% is characterized as light or faint staining, 10–40% as intermediate staining and greater than 40% as intense staining.

Upon sectioning, tissues from adult mice were differentially stained. The tissues were either stained lightly or faintly with an indistinguishable structure or pattern (percentage X-gal staining 1–10%), (Figs 5 and 6), medium or moderately stained with distinct pattern or cell type (percentage X-gal staining 10–40%), and heavily stained (percentage X-gal staining 40% and above). The staining percentages are based on image quantification of X-gal staining (Fig 6), as described in the materials and methods.

The faintly stained tissues include heart, pancreas, lymph nodes, skeletal muscles and thymus. Staining of the heart showed speckling over the myocardium that was not associated with the fascia, nuclei, or cytoplasm (Fig 5C). Interestingly, this pattern of staining was consistent in other forms of muscle, including skeletal muscle (from the quadriceps) (Fig 5H). In the pancreas, there was faint cytoplasmic staining in the islet cells (Fig 5I). In the lymph nodes, the staining was faint and diffused throughout the medulla, with more intense staining at the cortex, particularly in the perifollicular and sinus regions (which house macrophages and histiocytes) (Fig 5G).

The organs that were more moderately stained included the cerebral cortex, skin, spleen, retina, liver, lungs and small intestine. In the brains, the cerebral cortex (particularly the pyramidal cells (Fig 5A)), the cerebellar Purkinje cells and the hippocampus showed the most intense promoter activity (data not shown). There was light staining in the pyramidal tracts and the thalamus (data not shown). In the skin, staining was seen in the basal epithelial cells (Fig 5J). In the spleen, cytoplasmic staining could be seen in the marginal zone regions (possibly macrophages), and little to no staining was seen in the periarticular lymphocyte area (Fig 5L). Staining of sectioned eye revealed *Abcf1* promoter activity in the extraorbital muscles (data not shown), ciliary body (data not shown), and retina (Fig 5B). Within the retina, the ganglion cells appeared to be the most intensely stained. There was also intermediate to strong staining in both the inner and outer nuclear layers. There was virtually no staining in the cornea and sclera (data not shown). In the *Abcf1*^*+/-*^ liver sections, there was notable cytoplasmic staining in the peri-venular regions (Fig 5E). Sectioning and staining of the lungs showed slight *Abcf1* promoter activity in the respiratory epithelium, and more distinct or stronger staining in the bronchiolar (Fig 5F) and bronchi epithelium. The small intestine sections showed cytoplasmic staining of the mucosal epithelium as well in the lacteals (Fig 5K).

The highly stained areas included the kidney and testis. The kidney sections showed cytoplasmic staining in the proximal convoluted tubules and some staining in the glomerular apparatus (Fig 5D). The testis showed staining in the germinal epithelial cells and in the spermatozoa (Fig 5N). In all, the *Abcf1* promoter was found to be active in all organs and tissues tested. The activity, though, was different in the various tissues and particularly in different subsets of cells within those tissues. These data, however, should be interpreted with some degree of caution, as it is possible that the truncated fusion proteins are unstable or lead to toxicity in certain cell types. These observations should be followed up with cell specific immunostaining and Western blot to definitively identify the cell subsets that contain the highest levels of β-geo expression.

## Discussion

ABCF1 has been shown to be a key regulator of the DNA sensing pathway and regulates cytosolic DNA and retroviral infection though its exact molecular function remains unknown[5]. The *Abcf1* gene is induced by TNFα and functions in protein translational initiation and elongation [10]. In order to study ABCF1 in development and disease, we created a knockout mouse model, based on a single gene trap insertion in the *Abcf1* gene in embryonic stem cells. This construct further allowed lineage tracing of the endogenous *Abcf1* promoter by following the expression of a reporter gene. The gene trap cassette was inserted within the intronic region between exon 7 and exon 8 in the *Abcf1* gene, disrupting its ATP-binding activity. Heterozygous mice appeared to develop normally; however, embryos homozygous for the gene trap cassette died shortly after implantation, indicating that the loss of the ABCF1 protein is lethal in mice at a very early stage of development.

Previous studies showed that ABCF1 interacts with the eIF2 protein, which is a key member of the translation initiation machinery [7, 24]. ABCF1 is thought to bind to eIF2 through residues located in the N-terminal region and enhance the binding of eIF2 to Met-tRNA [10]. RNA interference experiments in Hela cells showed that a reduction in ABCF1 protein correlated with a reduction in general protein synthesis, as assayed through translation of reporter genes [7, 24]. Since mRNA translation is a fundamental process of life, any interference in this pathway should not be well tolerated in cells. This is also shown by the inability to produce *Abcf1*^*-/-*^ mice. Provocatively, *Abcf1*^*-/-*^ blastocysts can be produced and survive until implantation. This may be explained by the presence of long-lived maternal ABCF1, which is present up to the post-implantation stage of development, similar to the Argonaute 2 gene, which is a member of the RNA-induced silencing complex (RISC) complex [25]. Another explanation is that the N-terminal ABCF1-eIF2 association is sufficient for early embryo translation while the nucleotide binding domains have another distinct function, possibly in the control of translation. Since the XK097 gene trap is located between exon 7 and 8 of the *Abcf1* gene in our knockout mice, the N-terminal 186 AA should be still expressed. As ABCF1 is known to associate with eIF2 and is thought to be responsible for the initiation of translation, the fact that blastocysts can survive without the presence of the ABC cassettes and that the *Abcf1* promoter is preferentially expressed in functionally diverse tissues suggests that there may be a unique role for the ABC cassettes other than in translation initiation.

By taking advantage of gene trap technology, we provide the first detailed analysis of the *Abcf1* promoter activity during embryogenesis and in adult mice. Gene trapping, through the random insertion of trap vectors into the mouse genome, is an invaluable tool for generating mutants. Not only does it disrupt normal gene expression, but it has the added benefit of putting reporter genes under the control of the endogenous promoter. This allows the promoter activity to be examined under physiological conditions.

We demonstrate that the *Abcf1* promoter is highly active during mouse embryogenesis. The *Abcf1* promoter was found to be actively transcribed in blastocysts, and the expression of one copy of the wild type *Abcf1* gene was necessary for blastocyst survival to the post-implantation stage. The *Abcf1*^*-/-*^ blastocysts had extensive promoter activity, and morphologically appeared to be more oblong in shape. The *Abcf1*^*+/-*^ blastocysts also had strong promoter activity but appeared to resemble the *Abcf1*^*+/+*^ control blastocysts phenotypically. The only known function of ABCF1 is in its interaction with eIF2 and its requirement in translation initiation [7, 10]. Based on our data, the ABC cassettes in the ABCF1 protein do not appear to be important in contributing to this function in embryogenesis up to the blastocyst stage (and probably for implantation), as they are not expressed in the *Abcf1*^*-/-*^ blastocysts. We do know, however, that the presence of the ABCF1 ABC cassettes is essential for embryogenesis past the implantation stage, as we were unable to generate *Abcf1*^*-/-*^ post-implantation stage embryos. We gained some insight into the function of ABCF1 by examining the *Abcf1*^*+/-*^ embryos and mice. The *Abcf1* promoter was highly active in the *Abcf1*^*+/-*^ embryos throughout embryogenesis. Sectioning of 11.5 dpc embryos revealed strong staining in all tissues, particularly in regions known to be actively proliferating and differentiating. Interestingly, although the promoter activity appeared strong, 100% normal ABCF1 expression was not required for development, since *Abcf1*^*+/-*^ embryos, as well as adult mice, appear phenotypically normal when compared with their wild-type littermates. One explanation for this phenomenon may be that the N-terminal region of ABCF1 (which was previously found to interact with eIF2) in the truncated protein is adequate for normal translation, but there are also developmental roles that require the full protein.

In adult mice, the *Abcf1* promoter was found to be active in all organs and tissues tested, which was supported by the northern blot data. There were differences, however, in the promoter activity within different types of cells or regions within tissues and organs. There were also similarities between different tissues in terms of cell types with prominent staining. For example, within spleens and lymph nodes, staining appeared to be primarily in the regions surrounding the white pulp and the lymph node follicles. Both areas are known to contain macrophages. In the thymus, macrophage-like cells also appeared to be stained. Interestingly, mice that lack TNF cytokines, various transcription factors (*e*.*g*. NF-κB, rel), and chemokines tend to exhibit defects in marginal zone macrophage differentiation and positioning [26]. Interestingly, the *Abcf1* promoter was also found to be active in the eye and brain. These are organs, however, that are also to some degree actively regrowing or turning over cells in adult mice. The retina contains two different populations of monocyte-derived cells that are important in normal tissue homeostasis as well as inflammation [27]. These monocytic cells are known to turnover completely in 6 months, as shown by bone marrow transplantation experiments in mice expressing EGFP [27]. In the brain, GCN2 through its control of eIF2α phosphorylation, is known to be important in regulating synaptic plasticity associated with learning and memory [28]. Finally, the pattern of staining appeared to be consistent in specific types of muscle cells in the cardiac muscle, smooth muscle of the capillaries, and skeletal muscle. The pattern, however, was not associated with any particular cell type or structure, and thus cannot be associated with any particular function.

In conclusion, this study provides insight into the control of the *Abcf1* gene during embryogenesis and in adult tissues. Identifying the compartments that contain the highest level of promoter activity gives us an indication of which specific cell or tissue types may be affected by deficiency in *Abcf1* and facilitate the elucidation of the molecular function of *Abcf1*.
